# Antimicrobial and Antibiofilm Activity of a *Lactobacillus reuteri* SGL01, Vitamin C and Acerola Probiotic Formulation Against *Streptococcus mutans* DSM20523

**DOI:** 10.3390/biom16010158

**Published:** 2026-01-15

**Authors:** Adriana Antonina Tempesta, Gaia Vertillo Aluisio, Federica Di Gregorio, Roberta Lucia Pecora, Maria Lina Mezzatesta, Viviana Cafiso, Eleonora Chines, Giovanni Barbagallo, Maria Santagati

**Affiliations:** 1 Department of Biomedical and Biotechnological Sciences (BIOMETEC), Section of Microbiology, University of Catania, 95123 Catania, Italy; adriana.tempesta@unict.it (A.A.T.); gaia.vertilloaluisio@unict.it (G.V.A.); fededigrego@gmail.com (F.D.G.); roberta.pecora@phd.unict.it (R.L.P.); mezzate@unict.it (M.L.M.); v.cafiso@unict.it (V.C.); eleonora.chines01@universitadipavia.it (E.C.); 2 Department of Public Health, Experimental, and Forensic Medicine, University of Pavia, 27100 Pavia, Italy; 3 Centro Odontoiatrico Mediterraneo in Agreement with NHS, Private Dental Practice, 95123 Catania, Italy; giobar1201@gmail.com

**Keywords:** *Streptococcus mutans*, *Lactobacillus reuteri*, probiotic formulation, vitamin C, acerola, antimicrobial activity, antibiofilm activity

## Abstract

Dental caries is a multifactorial chronic infectious disease that impacts healthcare costs globally, caused by alterations of the plaque microbiome and proliferation of cariogenic *Streptococcus mutans*. Treatments targeting *S. mutans*, such as alternative strategies using probiotics, might be effective in preventing the development of dental caries. In this study, the probiotic formulation of *Lactobacillus reuteri* SGL01, vitamin C, and acerola was tested against *S. mutans* DSM20523. Antimicrobial activity was assessed by deferred antagonism and spot-on-lawn assays for *L. reuteri* SGL01. MIC and MBC of *L. reuteri* SGL01 cell-free supernatant (CFS), vitamin C, and acerola were determined with the microdilution method. Time–kill assays determined the bactericidal kinetics for each compound. The checkerboard method was used to evaluate the potential synergistic activity of CFS–vitamin C or CFS–acerola at scalar dilutions from 1 to 8X MIC. Lastly, antibiofilm activity was tested for each compound. Antimicrobial activity of *L. reuteri* SGL01 was first assessed by classic methods. MIC and MBC values differed for one dilution for all compounds, with values of 25% and 50% for CFS, 9.3 mg/mL and 18.7 mg/mL for vitamin C, and 18.7 mg/mL and 37.5 mg/mL for acerola, respectively. Moreover, time–kill assays confirmed the bactericidal activity at different timepoints: 4 h for CFS, 6 h for vitamin C, and 24 h for acerola. The fractional inhibitory concentration index (FICI) showed indifference for all combinations, and for associations tested at 2, 4, and 8XMIC. *S. mutans* biofilm production was impaired for all components, with stronger activity by vitamin C and acerola at lower concentrations. The probiotic formulation containing *L. reuteri* SGl01, vitamin C, and acerola extract exerts a bactericidal effect, especially strong for the CFS, as well as antibiofilm activity. Thus, the combination of these three components could be advantageous for their complementary effects, with use as a novel treatment against the development of dental caries by *S. mutans*.

## 1. Introduction

*Streptococcus mutans* is widely recognized as one of the main causative agents of human dental caries, primary due to its key virulence factors, such as its ability to form biofilms and its high acidogenic potential [1]. By fermenting dietary carbohydrates, particularly simple sugars, *S. mutans* produces organic acids that lower the local pH, leading to demineralization of the tooth surface and subsequent development of carious lesions. Moreover, the strong biofilm-forming capacity of *S. mutans* plays a pivotal role in the establishment of chronic, treatment-resistant infections, rendering *S. mutans* a clinically relevant pathogen in dental fields [2]. Biofilm formation improves bacterial survival by providing protection against antibiotics, immune responses, and environmental stresses such as nutrient limitation, temperature changes, and oxygen variability. This adaptive strategy makes biofilm-associated infections particularly difficult to treat, especially when associated with medical devices [3,4]. However, dental caries is a multifactorial disease resulting from the dysbiosis of oral microbiota, involving a complex interaction of microbial, host, and environmental factors. Repeated exposure to excessive dietary carbohydrates promotes an imbalance in dental plaque, lowering pH and favoring the proliferation of acidogenic and aciduric bacteria, thereby disrupting the homeostatic equilibrium of the resident oral biofilm [1]. Standard strategies to manage this condition focus on reducing the bacterial load through chlorhexidine and mechanical plaque removal; however, such approaches may be associated with adverse effects including increased tartar formation, mucosal irritation, and dental staining [5,6].

Recent studies have highlighted the beneficial effects of probiotics as a novel strategy for maintaining the oral ecosystem and preventing oral diseases, including dental caries [7]. By definition, probiotics are live microorganisms that when consumed in adequate amounts can confer health benefits to the host. Among these, lactobacilli (LBs), which are part of the oral microbiota, possess probiotic potential and are associated with maintaining oral health through antimicrobial effects [8,9]. Several studies have reported that LB strains exhibit significant inhibitory activity against *Streptococcus mutans* [10,11,12]. Among these species, *Lactobacillus reuteri*, part of the healthy oral microbiota, displays antibacterial properties due to its production of reuterin—an antimicrobial compound generated during anaerobic glycerol fermentation—and reutericyclin [13,14,15] and bacteriocins such as reutericin [16] that can inhibit or interfere with pathogenic strains, thereby contributing to a balanced microbiota. Moreover, *L. reuteri* exerts numerous beneficial effects on individual health thanks to its ability to prevent colonization of pathogenic bacteria, remodel the commensal microbiota composition, and modulate the host immune system [17,18]. Recent studies have investigated the adjunctive use of *L. reuteri* in peri-implant disease management. A prospective clinical study on patients with peri-implant mucositis evaluated the effect of a 30-day regimen of *L. reuteri* tablets (one daily), demonstrating that after three months the study group showed a significant reduction in plaque index (PI) and bleeding on probing (BOP) around both implants and natural teeth [19]. Similarly, *L. reuteri* supplementation may contribute to reductions in probing pocket depth (PPD), particularly in peri-implant mucositis [20,21].

Vitamin C (ascorbic acid) and acerola extract have also been proposed as adjunctive therapies for peri-implant conditions, with evidence suggesting potential improvements in clinical outcomes such as PPD and BOP. Vitamin C plays a crucial role in collagen synthesis and protection against reactive oxygen species (ROS), acting as a potent antioxidant and contributing to the reduction of oxidative stress [22]. Acerola (*Malpighia emarginata*) is a tropical fruit exceptionally rich in phytochemicals and widely recognized as one of the most abundant natural sources of vitamin C [23]. The high ascorbic acid content contributes to its anti-inflammatory properties, while carotenoids such as β-carotene further enhance its anti-inflammatory potential and may modulate gut microbiota composition [24].

Moreover, studies conducted on a gut microbiota model evinced the combinatory positive activity of acerola and probiotics in modulating the microbial composition and metabolism, since the dry extract of acerola contains a high amount of carbohydrates, proteins, fibers, and phenolic compounds that can contribute to the survival of probiotics [23]. In vivo studies on rats fed a high-fat diet supplemented with acerola showed reductions in *Escherichia coli*, *Enterococcus* spp., and Enterobacteriaceae, accompanied by increased levels of *Lactobacillus* spp. and *Bifidobacterium* spp. [22]. In vitro studies have also demonstrated the antimicrobial activity of vitamin C and acerola against oral pathogens, particularly *S. mutans* and periodontal pathogens [25,26]. Although no in vitro studies to date have specifically evaluated the effects of acerola on oral streptococci such as *S. mutans*, acerola extracts have shown antibacterial activity against other Gram-positive bacteria, including *Staphylococcus aureus*, in agar diffusion assays [27].

Considering evidence supporting the beneficial effects of oral probiotics and adjuvants in modulating the oral microbiota and exerting antimicrobial activity against various pathogens, the present study evaluated the antibacterial and antibiofilm efficacy of the individual components of a commercially available oral probiotic formulation (PAROSAN^®^)—*L. reuteri* SGL01, vitamin C, and acerola extract—as well as their potential synergistic action against *S. mutans* DSM 20523.

## 2. Materials and Methods

### 2.1. Bacteria Strains and Culture Conditions

*S. mutans* DSM 20523 was grown in brain heart infusion broth (BHI) (Oxoid, Basingstoke, UK) and on BHI agar plates at 37 °C for 24 h with 5% of CO_2_. *L. reuteri* SGL01 was grown in de Man, Rogosa, and Sharpe (MRS) agar (Oxoid, Basingstoke, UK) and incubated for 48 h at 37 °C under anaerobic conditions, using GasPakEZ Gas Generating Pouch Systems (BD Diagnostics, Franklin Lakes, NJ, USA).

### 2.2. Probiotic Formulation

The probiotic formulation (PAROSAN^®^; Nutraceutica s.r.l., Bologna, Italy) used in this study contains 30% of vitamin C, 10% of *L. reuteri* SGL01 (10 MLD), and 1% of dry acerola extract (standardized to 50% vitamin C). *L. reuteri* SGL01, vitamin C, and acerola were tested for their antimicrobial activity.

### 2.3. Deferred Antagonism and Agar Spot Assays

The inhibitory activity of *L. reuteri* SGL01 was first tested against the indicator strain *S. mutans* DSM 20523 by the deferred antagonism test, as previously described [28], inoculating *L. reuteri* SGL01 on MRS agar supplemented with 0.1% of calcium carbonate to reduce lactic acid-related inhibitory effects, and incubated for 48 h at 37 °C under anaerobic conditions to examine the interference zones of the indicator strain.

For the agar spot assay, 5 µL of *L. reuteri* SGL01 broth culture was spotted on the surface of MRS soft agar plates (1.2%) and incubated anaerobically for 48 h at 37 °C. An overnight culture of indicator strains (approximately 10^7^ CFU/mL) was mixed with BHI soft agar (0.7%) and poured over the plate where lactobacilli were spotted, as previously described [29]. Antagonistic activity was assessed by measuring the diameters of inhibition zones and expressed as follows: no inhibition (−); diameter between 1 and 3 mm (+); diameter between 3 and 6 mm (++); diameter between 6 and 10 mm (+++); and diameter > 10 mm (++++).

### 2.4. Lactobacillus reuteri SGL01 Cell-Free Supernatant Preparation

Cell-free supernatant (CFS) of *L. reuteri* SGL01 was prepared as previously described [29]. SGL01 was inoculated in MRS broth (Oxoid, Basingstoke, UK) and incubated for 48 h at 37 °C under anaerobic conditions. Broth culture was centrifuged at 7000× *g* for 30 min at 4 °C to remove pelleted cells and filtered sterilized with a 0.22 µm bottle filtration unit (Merck Millipore, Darmstadt, Germany). pH values of CFS were measured by a pH meter (pH 50+DHSBenchtop pH Meter, Via della Meccanica, Carpi, Italy).

### 2.5. In Vitro Susceptibility Tests by MIC and MBC

Minimum inhibitory concentration (MIC) values of *L. reuteri* SGL01 CFS, vitamin C, and acerola were determined by the standard broth microdilution method using an inoculum of approximately 5 × 10^5^ CFU/mL according to CLSI guidelines [30]. BHI broth was used as the medium for MIC determination. Serial concentrations were tested for CFS (50% to 0.024%), vitamin C (150 mg/mL to 0.07 mg/mL), and acerola (300 mg/mL to 0.5 mg/mL). Microdilution plates (96-well) were incubated at 37 °C with 5% CO_2_ for 24 h.

Minimal bactericidal concentration (MBC) of all constituents was determined by CLSI broth microdilution methodology. A volume of 100 μL of broth from each well with no visible growth after 24 h was sub-cultured on BHI agar plates to determine the 99.9% kill endpoint. *S. mutans* count plates were then incubated at 37 °C with 5% CO_2_ for 24 h. All experiments were performed in triplicate. The MBC was defined as the lowest concentration of substance producing a colony count <0.1% of the initial inoculum. Given the starting inoculum (~5 × 10^5^ CFU/mL), this threshold corresponds to 5 × 10^2^ CFU/mL, i.e., approximately ≤50 colonies when plating 100 µL.

An antimicrobial agent can be considered bactericidal when the MBC-to-MIC ratio is <4, as previously reported [25].

### 2.6. Time–Kill Assay

The bactericidal effects of CFS, vitamin C, and acerola were evaluated at MIC values 1X by time–kill curve experiments using a 20 mL tube containing BHI broth with an initial inoculum of 10^6^ CFU/mL. The kinetic growth of the strains was tested at different time points (0, 4, 6, 8, and 24 h). At each time point, viable counts (CFU/mL) were determined by plating 100 µL of on BHI agar (limit of detection, LOD = 10 CFU/mL for 100 µL).

Bactericidal activity was defined as a decrease of at least 99.9% (≥3 log_10_) from the initial inoculum within 24 h [28]. The results were expressed as the mean value of log_10_ CFU/mL ± standard deviation from three independent assays.

### 2.7. Broth Microdilution Checkerboard Method

Synergy or antagonism of probiotic constituents against *S. mutans* DSM 20523 was assessed by the checkerboard method, testing the antimicrobial combinations of CFS and vitamin C or CFS and acerola. Using a 96-well plate, a two-fold dilution of the freshly prepared antimicrobial at different concentrations was dispensed in a checkerboard array and inoculated with a test organism to yield the appropriate density 5 × 10^5^ CFU/mL in a final volume of 0.1 mL, and then incubated at for 24 h at 35 °C in ambient air [31]. Concentrations from 1× to 8× MIC were tested for vitamin C and acerola, while CFS was tested starting from 1× MIC. A well with no antimicrobial compounds was used as the positive control. After incubation, wells were inspected visually, and turbidity was considered indicative of growth. The resulting checkerboard contained each combination of two antimicrobial compounds, with wells that contained the highest concentration of each antimicrobial at opposite corners. According to the CLSI guidelines for broth microdilution, the MIC was defined as the lowest concentration of the antimicrobial that completely inhibited the growth of the organism, as clearly observable upon visual examination. The fractional inhibitory concentration index (FICI) was calculated as follows:

FICI = (MIC A combination/MIC A single) + (MIC B combination/MIC B single)

The combination was considered synergistic when the ΣFIC is ≤0.5, additive when the ΣFIC is >0.5 to ≤1, indifferent when the ΣFIC is >1 to ≤4, and antagonistic when the ΣFIC is >4 [31].

### 2.8. Biofilm Formation of S. mutans DSM20523

Biofilm of *S. mutans* DSM20523 was obtained as previously described [32], with some modifications. A preculture of *S. mutans* was diluted to reach a 10^6^ CFU/mL inoculum in BHI + 2% of sucrose (BHIS), then 200 µL of inoculum were dispensed in a 96-well flat-bottom microtiter plate in triplicate and incubated at 37 °C with 5% CO_2_ for 24 h. Free BHIS was used as the negative control. After incubation, the plate was decanted, washed three times with sterile saline, and left to air-dry. A total of 200 µL of 0.1% crystal violet was added to each well and incubated at room temperature for 15 min. After washing it thrice in distilled water, the plate was air-dried. A total of 200 µL of glacial acetic acid 33% was added to each well, left to rest for 10 min, then read at 600 nm with a BioTek Synergy H1 plate reader (Agilent Technologies, Santa Clara, CA, USA). The resulting OD_600_ values were analyzed as previously reported, comparing the OD_600_ values of control wells (not-inoculated medium) with the OD_600_ values of inoculated wells [32].

### 2.9. Inhibitory Activity on S. mutans DSM 20523 Biofilm

To test the antibiofilm activity of *L. reuteri* SGL01 CFS, vitamin C, and acerola, scalar dilutions of the antimicrobial agents starting from a 1:1 ratio were prepared in a 96-well flat-bottom microtiter plate, with a final volume of 100 µL per well as previously described [29]. Then, 100 µL of *S. mutans* inoculum in BHIS was added. For growth control, 200 µL of *S. mutans* in BHIS was dispensed. For negative control, BHIS was allotted. After incubation at 35 °C with 5% CO_2_ for 24 h, the plate was stained as aforementioned and OD_600_ values were read accordingly. All conditions were performed in triplicate.

To assess the inhibition ability of each antimicrobial, OD_600_ values of treated wells were normalized with the following formula:

$$\mathrm{Percentage}\;\mathrm{of}\;\mathrm{biofilm}=\frac{T-B}{C-B}\;\times 100$$

where C represents the OD_600_ values for control wells (untreated *S. mutans* biofilm), T is the OD_600_ values of treated wells, and B indicates the OD_600_ values of the negative control. All experiments were performed in at least three independent assays. Statistical analysis with multiple comparisons was performed using the two-way ANOVA method.

## 3. Results

### 3.1. Anti-Streptococcal Activity of L. reuteri SGL01

The deferred antagonism and agar spot assays showed the inhibitory activity of *L. reuteri* SGL01 against *S. mutans* DSM 20523.

**Figure 1:**
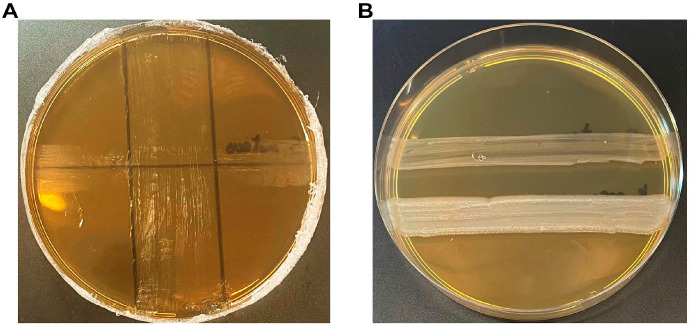
Deferred antagonism test. (**A**) Inhibition of *S. mutans* DSM 20523 (transversal line) growth across *L. reuteri* SGL01 line (vertical line). (**B**) Control growth of *S. mutans* DSM 20523 on MRS agar.

In particular, the deferred antagonism revealed the possible production of metabolites that inhibit *S. mutans* growth, minimizing the acid-related effect by supplementing MRS agar with calcium bicarbonate (Figure 1). This activity could be associated with the production of bioactive metabolites such as bacteriocins [29].

**Figure 2:**
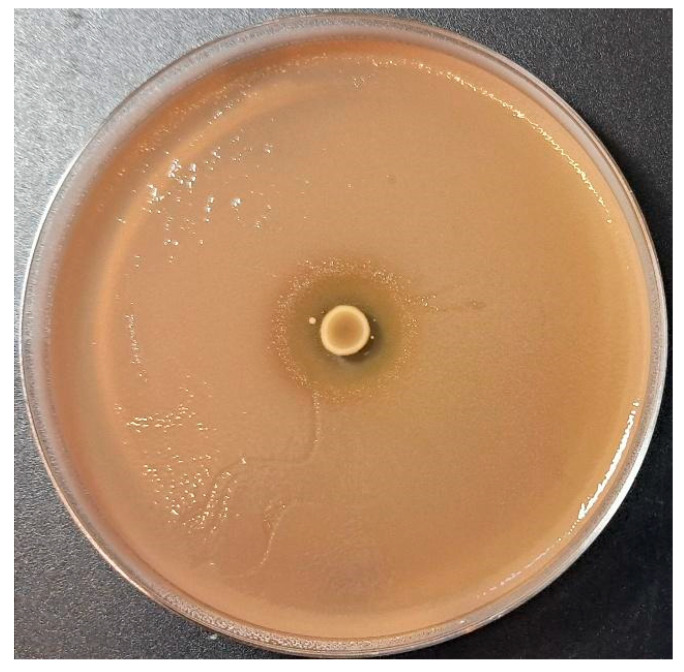
Agar spot test for *S. mutans* DSM 20523. Strong antagonistic activity of *L. reuteri* SGL01 was confirmed with a diameter of inhibition zone > 10 mm.

Moreover, an agar spot test was used to quantify the antimicrobial activity exerted by *L. reuteri* SGL01, which showed the strongest growth inhibition against *S. mutans* with inhibition zone diameters > 10 mm (+ + + +) (Figure 2).

### 3.2. Antibacterial Activity of Probiotic Components by MIC and MBC

In vitro antibacterial activity of *L. reuteri* SGL01 CFS, vitamin C, and acerola against the reference strain *S. mutans* DSM 20523 are reported in Table 1. The MIC and MBC values were expressed as % *v*/*v* and mg/mL. The probiotic components tested showed antibacterial activity against *S. mutans* DSM 20523, with MIC values equal to 25% for CFS and values of 9.3 mg/mL and 18.7 mg/mL for vitamin C and acerola, respectively. The MBC values were one dilution higher than the MICs for CFS (50%), as well as for vitamin C and acerola (18.7 mg/mL and 37.5 mg/mL, respectively). In the endpoint MBC assay, recoverable growth was still observed upon subculture from the MIC well, whereas the 99.9% kill criterion was met at the next two-fold concentration (2× MIC).

### 3.3. Bactericidal Effect by Time–Kill Curves

**Figure 3:**
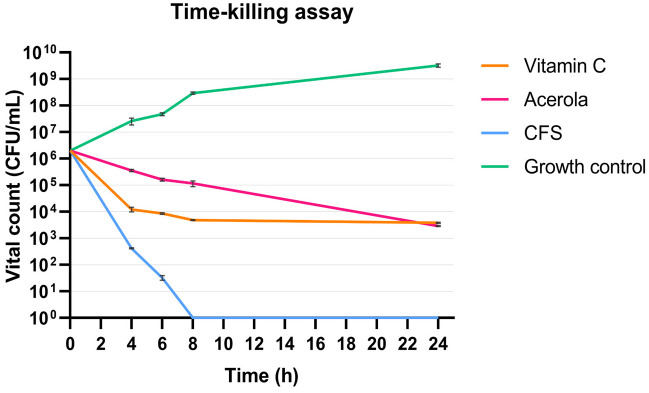
Time–kill curves of probiotic components against *S. mutans* DSM 20523. Differences between treated conditions and growth control highlighted the bactericidal activity of the three different substances, as shown by the plotted data expressed as the mean value of CFU/mL ± standard deviation. The limit of detection (LOD) was 10 CFU/mL. Values <LOD were displayed at the baseline for graphical representation.

Time–kill kinetics studies of CFS, vitamin C, and acerola against *S. mutans* DSM 20523 are shown in Figure 3. Bactericidal activity was defined as a decrease in the initial inoculum by at least 3 log_10_ CFU/mL. The bactericidal activity was observed at T4 (4 h) for CFS, at T6 (6 h) for vitamin C, and at T24 (24 h) for acerola. Overall, all tested compounds showed bactericidal activity (99.9% of initial inoculum) at T24, but the bactericidal kinetics were faster for CFS, with a reduction of 3 log_10_ CFU/mL at T4. From T8 (8 h) to T24 (24 h), viable counts for CFS were below the limit of detection (<LOD; 10 CFU/mL). Conversely, vitamin C exerted its bactericidal activity at T6 with a 3 log_10_ reduction, which was maintained throughout T8 and T24. Lastly, the kinetics of acerola were slower and showed only a 1 log_10_ reduction between T4 and T8, with a 3 log_10_ reduction at T24.

### 3.4. Antimicrobial Interactions by Checkerboard Assays

The results for the combinations CFS–vitamin C and CFS–acerola against *S. mutans* DSM 20523 are reported in Table 2. Checkerboard assays were performed to evaluate potential interactions between *L. reuteri* SGL01 CFS and either vitamin C or acerola. The combinations tested at 1×, 2×, 4×, and 8× MIC yielded FICI values ranging from 1.02 to 1.10 for the CFS–vitamin C association and from 1.06 to 1.15 for the CFS–acerola association. According to established interpretive criteria (ΣFIC > 1 to <4), all tested combinations fell within the indifference range, indicating neither synergistic nor antagonistic interactions. Increasing the concentrations of the combined agents did not significantly modify the interaction profiles, as FICI values remained consistently close to 1 across all tested levels.

### 3.5. Antibiofilm Activity

**Figure 4:**
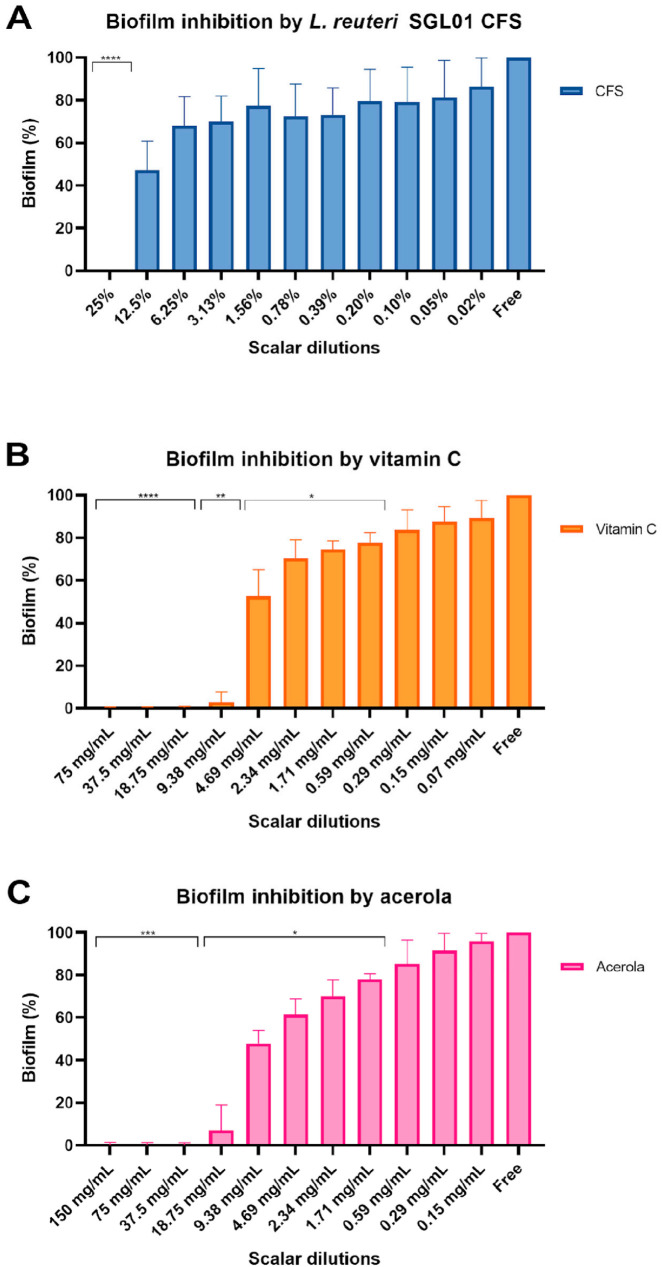
Biofilm of *S. mutans* DSM 20523 expressed in percentage. OD_600nm_ values of treated wells were normalized using control values. (**A**) Antibiofilm activity of *L. reuteri* SGL01 CFS. (**B**) Antibiofilm activity of vitamin C. (**C**) Antibiofilm activity of acerola. Statistical significance was expressed as (* *p* ≤ 0.05, ** *p* ≤ 0.01, *** *p* ≤ 0.001, **** *p* ≤ 0.0001).

The ability of *S. mutans* DSM 20523 to produce biofilm, evaluated by crystal violet assay, showed that *S. mutans* was a strong biofilm producer with a mean OD_600_ value of 2.93 ± 0.19 (SD). The treatment of biofilm *S. mutans* with the antimicrobial compounds showed different effects depending on the probiotic components, as reported in Figure 4. For CFS, the antibiofilm activity was exerted mostly at a concentration of 25%, with a complete lack of biofilm production compared to the control (*p* value < 0.0001). At lower concentrations CFS showed weaker activity, only slightly affecting the biofilm production. Conversely, both vitamin C and acerola had stronger antibiofilm activity, with a complete lack of biofilm production at higher concentrations (*p* value < 0.0001 for vitamin C and <0.001 for acerola) and an impaired production at lower values ranging from 4.69 mg/mL to 0.59 mg/mL for vitamin C (*p* value < 0.05) and 18.75 mg/mL to 1.71 mg/mL for acerola (*p* value < 0.05). Interestingly, the antibiofilm activities of vitamin C and acerola were maintained at lower concentrations than their respective MIC values.

## 4. Discussion

Dental caries remains a major global health concern that can affect the quality of life of patients [33], but also be related to systemic health as poor oral health is linked to systemic diseases [34,35]. The primary cause of dental caries is *Streptococcus mutans* and its ability to colonize and disrupt the local microbiome, and if untreated chronic dental caries can also lead to a persistent inflammatory response [35]. Thus, probiotic strategies can be an innovative preventive approach to use in the oral cavity, due to their potential to restore balance and inhibit cariogenic bacteria [36]. Several studies have reported beneficial effects exerted by probiotic lactobacilli in the oral cavity, showing reduction of *S. mutans* counts and improvement of oral microbial balance [8,37]. In particular, antagonistic mechanisms may include adhesion to the oral mucosa and competition with pathogens for colonization niches, co-aggregation with pathogens, and production of inhibitory metabolites (postbiotics) that impair pathogen growth and biofilm formation [9,13,38].

Our study aimed to characterize the antimicrobial activity of a probiotic formulation containing some excipients that could have antimicrobial activity targeting *S. mutans*, specifically *Lactobacillus reuteri* SGL01, vitamin C, and acerola. In particular, the activity of the strain *L. reuteri* SGL01 was tested against *S. mutans* DSM 20523, confirming that this probiotic strain has the ability to inhibit the cariogenic *S. mutans* by deferred antagonism and agar spot assays, as previously described [28,29]. The former assay is crucial as it detects the antimicrobial activity exerted by *L. reuteri* SGL01, minimizing the acidic activity of this strain by supplementing MRS agar with calcium carbonate; conversely, the latter assay provides a quantitative assessment of the inhibitory activity of *L. reuteri* SGL01, distinguishing between weak and strong effects. These results are in accordance with clinical studies that support the relevance of *L. reuteri* in oral health. In preschool children, daily consumption of *L. reuteri* lozenges significantly reduced salivary mutans streptococci counts, indicating a measurable caries-preventive effect in vivo [39]. Similar results were observed in young adults, where *L. reuteri* lozenges produced a marked reduction in *S. mutans* levels after a short intervention period [40].

Accordingly, when the cell-free supernatant (CFS) of *L. reuteri* SGL01 was tested against *S. mutans* DSM 20523, MIC (25%) and MBC (50%) values were obtained and time–kill curves showed how the bactericidal activity of CFS was reached after only 4 h, with stronger and faster results than the other tested components (vitamin C and acerola). Furthermore, a bactericidal effect on the initial inoculum was observed after 24 h, as viable counts from T8 (8 h) through T24 (24 h) were below the limit of detection (<LOD) under our plating conditions. Our findings are also in agreement with previous studies showing that the CFSs of *L. reuteri* strains, such as ATCC 23272 [12] and AN417 [41], exhibit antibacterial activity and significantly reduce *S. mutans* biofilm biomass.

*L. reuteri* SGL01 CFS was also tested for biofilm inhibition as *L. reuteri* has the ability to disrupt the acid tolerance response (ATR) of several oral bacteria, directly weakening their ability to survive under low-pH conditions typical of cariogenic biofilms [42]. The CFS prevented biofilm formation by *S. mutans* DSM 20523, with a complete effect at higher concentration (25%) but also an intermediate inhibition at lower concentrations (12.5% to 3.13%). The disruption of ATR, which is essential for *S. mutans* aciduricity and competitiveness within dental plaque, could be involved in the potent antibacterial and antibiofilm activity of the SGL01 CFS observed in our assays. These results are consistent with evidence showing that CFSs of various *Lactobacillus* strains, including *L. reuteri*, can inhibit *S. mutans* growth and disrupt biofilm structure through the combined action of organic acids, bacteriocin-like molecules, and other secreted metabolites [43,44].

Beyond probiotic-derived metabolites, our findings also highlight the relevant antimicrobial and antibiofilm properties of vitamin C and acerola extract against *S. mutans.* The MIC and MBC values obtained in this study confirm the bactericidal activity of both vitamin C and acerola, a dried extract that contains 50% of vitamin C along with other components. Furthermore, these findings are in agreement with prior studies showing that ascorbic acid can directly inhibit the growth, metabolic activity, and biofilm formation of *S. mutans* clinical isolates. Notably, Eydou et al. reported a MIC of 9.3 mg/mL for vitamin C [25], which is consistent with the value observed in our study, further supporting its direct antibacterial activity. Likewise, acerola-derived formulations have demonstrated antimicrobial potential in oral disease models, including periodontitis, further supporting its translational value in oral care application [26].

Moreover, time–kill assays evinced the bactericidal activity of vitamin C and acerola, although this activity was observed at later timepoints compared to *L. reuteri* SGL01 CFS (6 and 24 h, respectively). These time differences between vitamin C and acerola might depend on acerola composition, which comprises several phytonutrients like carotenoids, phenolics, anthocyanins, and flavonoids [45]. Under our experimental conditions, neither vitamin C nor acerola reduced viable counts below the limit of detection, highlighting the more rapid and pronounced killing kinetics observed for *L. reuteri* SGL01 CFS.

Conversely, both vitamin C and acerola significantly impaired biofilm formation, even at sub-MICs, in a similar manner for both compounds, suggesting that ascorbic acid could interfere with early adhesion or extracellular polymeric substance (EPS) synthesis [46]. This activity is particularly relevant for prevention, as targeting the initial phases of biofilm development represents a key strategy for limiting *S. mutans* colonization and cariogenicity. Moreover, the observed results highlight how these two components of the probiotic formulation may play a stronger role in the inhibition of biofilm formation, targeting this process which is essential for pathogenicity of *S. mutans*, whereas *L. reuteri* SGL01 may be more active on planktonic cells and their viability.

Lastly, FIC analysis showed that the combinations of *L. reuteri* SGL01 CFS and vitamin C and CFS and acerola did not show synergistic activity, with FICI values consistently within the indifference range [47]. Lack of synergy can occur even when components act through different pathways, for instance when the dominant effect is driven by one component (e.g., rapid bactericidal activity of CFS and/or may include pH-related effects), leaving limited additional benefit from co-administration within the concentration range explored in MIC-based assays. Moreover, synergy metrics derived from MIC endpoints may not capture complementary, non-lethal effects that are relevant for biofilm prevention [47]. Accordingly, we emphasize that the formulation shows relevant antimicrobial and antibiofilm activity with complementary contributions from its components, while no evidence of synergy was observed under the tested in vitro conditions.

## 5. Conclusions

In conclusion, the combination of probiotics, vitamin C, and acerola shows that the antimicrobial and antibiofilm activities of vitamin C and acerola effectively complement the rapid bactericidal action of *L. reuteri* SGL01. Importantly, the absence of antagonism among these components supports their combined use within a multimodal probiotic-based approach. Such an approach may enhance ecological control within the oral cavity and hold translational potential for the development of integrated anti-cariogenic formulations.

### Limitations

Although a monospecies *S. mutans* model is appropriate for caries-related investigations, it does not fully reproduce the complexity of oral biofilms. In addition, all assays were conducted using a single reference strain, which may not reflect the variability observed among clinical isolates. Lastly, the bioactives present in the CFS were not characterized, and further studies will be necessary to investigate which metabolites are involved in this antimicrobial activity.

## Figures and Tables

**Table 1:** Minimum inhibitory concentrations (MICs) and minimum bactericidal concentrations (MBCs) of probiotic components against *S. mutans* DSM 20523.

Substance	MIC	MBC
*L. reuteri* SGL01 CFS (% *v*/*v*)	25	50
Vitamin C (mg/mL)	9.3	18.7
Acerola (mg/mL)	18.7	37.5

**Table 2:** Results obtained with probiotic combinations by checkerboard method against *S. mutans* DSM 20523. Vitamin C and acerola were tested at 1×, 2×, 4×, and 8× MIC.

Fold-MIC Level	∑FIC CFS–Vitamin C	∑FIC CFS–Acerola	Activity ª
1×	1.02	1.08	I
2×	1.05	1.06	I
4×	1.08	1.11	I
8×	1.10	1.15	I

ª S, synergy; A, antagonism; I, indifference.

## Data Availability

The original contributions presented in this study are included in the article. Further inquiries can be directed to the corresponding author.

## Abbreviations

The following abbreviations are used in this manuscript:

ATR	Acid Tolerance Response
BHI	Brain Heart Infusion
BHIS	BHI + 2% Sucrose
CFS	Cell-free Supernatant
CFU	Colony Forming Unit
FIC	Fractional Inhibitory Concentration
FICI	Fractional Inhibitory Concentration Index
LOD	Limit of Detection
MBC	Minimum Bactericidal Concentration
MIC	Minimum Inhibitory Concentration
MRS	De Man, Rogosa, Sharpe
